# Author Correction: Continuous moulting by Antarctic krill drives major pulses of carbon export in the north Scotia Sea, Southern Ocean

**DOI:** 10.1038/s41467-021-22037-y

**Published:** 2021-03-05

**Authors:** C. Manno, S. Fielding, G. Stowasser, E. J. Murphy, S. E. Thorpe, G. A. Tarling

**Affiliations:** British Antarctic Survey, Natural Environment Research Council, Cambridge, CB3 0ET UK

**Keywords:** Biochemistry, Ecology, Biogeochemistry, Climate sciences, Ecology

Correction to: *Nature Communications* 10.1038/s41467-020-19956-7, published online 27 November 2020.

The original version of this Article contained an error in the Discussion, which incorrectly read ‘Assuming a krill population biomass of 379 Mt for the Southern Ocean, we estimate that the exuviae flux can contribute a seasonally averaged mean of 0.29 t C d−1 (SE 0.09; see Supplementary Methods).’ The correct version adds ‘M’ to the unit before ‘t C d-1’. The Supplementary Information file was also updated as one paragraph had many errors. These errors have been corrected in both the PDF and HTML versions of the Article.

## Supplementary information

Supplementary Methods

