# Supplementary material for: Continuous moulting by Antarctic krill drives major pulses of carbon export in the north Scotia Sea, Southern Ocean
Source: Nat Commun. 2020 Nov 27;11:6051. doi: 10.1038/s41467-020-19956-7 (PMC7699634; doi:10.1038/s41467-020-19956-7)
Supplement: Supplementary file 1 — Supplementary Information [file 41467_2020_19956_MOESM1_ESM.pdf]

Supplementary Information for Continuous moulting by Antarctic krill drives major pulses of carbon export in the north Scotia Sea, Southern Ocean by *Manno et al.*

Calculation of krill standing stock biomass from krill exuviae captured by the sediment trap

The following details the methods used to determine the depth integrated biomass of krill (g wet weight [WW] m^{-2}) in the vicinity of a mooring in the northern Scotia Sea (53° 47.90'S, 37° 55.99'W) using, as the primary data source, the exuviae of Antarctic krill captured by a moored-sediment trap over a 12 month period. The estimates represent a daily mean biomass over the period in which respective sediment trap bottles were open. Depending on the time of year, these opening periods were set to intervals of either 15 or 30 days (*d*).

The calculation can be broken down into 5 main elements: 1. determining the intermoult period (IMP), which also represents the number of randomly moulting krill required to produce one exuviae per day; 2. calculating the equivalent wet weight (WW) of a moulting krill; 3. estimating the spatial scale of capture of the sediment trap; 4. combining parameters 1 to 3 to derive mean krill biomass; and 5. making an assessment of reasonable upper and lower bounds for the mean biomass estimate.

1. Determination of intermoult period

1.1: Krill total length was estimated from uropod length following Miller (1983):

$$S1_{i,j} = 8.192 + 5.233 \cdot UL_{i,j} \qquad (1)$$

where *S1* is estimated standard body length (mm) and *UL* is measured uropod length (mm) from an exuvia, *i* is the specimen and *j* is the sediment trap bottle. The number of exuviae measured from each bottle (m_j) was a maximum of 100. For the majority of bottles, m_j encompassed all of the exuvaie collected (n_j), but was less than n_j in some others, which reached up to a maximum 280 exuviae (Supplementary information table 2.2).

1.2: The IMP per individual, $IMP_{i,j}$, was determined through applying equations provided by Tarling et al. (2006) where the parameter *L* in Tarling et al. was $S1_{i,j}$ and the parameter *T* in Tarling et al. was climatological sea surface temperature for the South Georgia region (Whitehouse et al. 2008) for the respective time period corresponding to sediment trap bottle *j*. It is also to be noted that, in using their equations, their parameter *d* must equal 5 as it refers to the duration of their incubation experiments. The Tarling et al. equations require sex and developmental stage to be known. This could not be determined from the exuviae extracted from the sediment trap samples. Therefore, estimates of $IMP_{i,j}$ for each of the four potential sex/developmental stages discriminated by Tarling et al. were made and an average across all four stages determined, to give $\overline{IMP}_{i,j}$. An average IMP was then derived for each bottle *j* as follows:

$$\overline{IMP}_j = \frac{\sum_{i=1}^{i=m} \overline{IMP_{i,j}}}{m_j} \qquad (2)$$

For the winter period (March to August), $\overline{IMP_{i,j}}$ was multiplied by 5, in line with the increase in winter IMP reported by Buchholz et al. (1989).

1.3: The average daily number of captured exuviae, e_j, is n_j/d_j, and the number of krill required to generate e_j is $\overline{IMP_j} \cdot e_j$.

2. The equivalent wet weight (WW) of krill

2.1: The WW (w, g) of an individual krill, i, caught within sediment trap bottle j, was estimated using the following equation, provided by (Kils 1981):

$$w_{i,j} = 1.58 \cdot 10^{-6} \cdot S1_{i,j} \quad (3)$$

2.2: G_j (g), the required WW of krill to generate e_j, can be calculated accordingly:

$$G_j = \frac{\sum_{i=1}^{i=m} \overline{IMP_{i,j}} \cdot w_{i,j}}{m_j} \cdot e_j \quad (4)$$

3. Spatial scale of capture by the sediment trap

We define the term spatial scale of capture as the distance between the sediment trap and the remote location from which a captured exuvia was released. Within this scale, all moulted exuviae have the potential to be captured by the sediment trap. Not all potential captures will be realised however, since a number of forces act on the sinking exuviae, moving them in and out of the capture zone, i.e. the zone which figuratively lies within the mouth area of the sediment trap (Supplementary information figure 1). How representative the number of captured exuviae are will depend on the distribution of krill within the spatial scale of capture (i.e. whether patchily or evenly distributed) and the time period of capture over which the results are integrated.

To calculate the spatial scale of capture, we followed the virtual transect principle, detailed in Brierley et al. (2006). This method uses horizontal velocity fields from a fixed point mooring to determine the remote origin of the water column observed within a set time period. It was also necessary to make the following further assumptions: (i) that the sinking speed of an exuvia was 1 cm.s^{-1} (Nicol and Stolp 1989), such that the maximum time taken to reach the sediment trap at 300 m depth was 8.3 h, and (ii) that the exuvia moved passively with prevailing horizontal ocean currents as it sank through the water column.

Prevailing horizontal velocity fields were measured by an upward looking acoustic Doppler current profiler (ADCP) situated above the sediment trap (187 m) during the period of deployment. The ADCP collected data in 15 minute ensembles and 8 m depth bins. Frequently, there were patches of water near the surface where data quality was reduced, probably due to lack of scatterers, and these were masked out of subsequent data processing. We considered only ensemble-bins in which all data were calculated from all four beams of the ADCP. The non-masked data were averaged through the resolved water column to produce depth-averaged u and v velocity components for

each 15 minute ensemble. These components were applied to determine the trajectory of a passive particle over an 8.5 h period (i.e. the time taken for an exuvia to sink to 300 m, rounded up to the nearest ADCP ensemble interval). Trajectories were started from local midnight, as the most likely time krill moult over the diel cycle (Tarling et al. 1999). The straight distance between the location of the sediment trap and the final location of the passive particle at 8.5 h was then determined for each day of the deployment to derive D_d (where D is distance in m and d is day). Values for D_d were subdivided according to sediment trap bottle period, j, and then analysed to derive values for the mean ($D_{j,mean}$), maximum ($D_{j,max}$) and minimum ($D_{j,min}$) straight distances travelled during period j. $D_{j,mean}$ ranged between 1000 and 1800 m, with a maximum outer range (maximum $D_{j,max}$) of ~4700 m and minimum outer range (minimum $D_{j,min}$) of ~80 m (Supplementary information table 2.1).

4. Mean estimate Antarctic krill biomass

To calculate a mean biomass for Antarctic krill, we assumed (i) that all krill were located in the top 50 m of the water column during nighttime (Tarling et al. 2009) and (ii) that the krill population was evenly distributed over the mean spatial scale sampled by the sediment trap (Scenario A in supplementary information figure 1). We considered the latter a reasonable assumption for calculating the mean biomass given that, over the 15 or 30 day sample interval, it is likely that a large number of swarms and more diffuse aggregations will be moving around and through the 1 to 2 km mean spatial scale of capture, evening out any shorter-term patchiness in distribution.

Under this scenario (Scenario A, supplementary information figure 1), the density of krill moulting in the zone of capture is representative of the wider situation. Hence, the effective sampling area would be equivalent to mouth area of the sediment trap (0.5 m^2) because the density of moulting krill directly above the sediment trap, or elsewhere within the spatial scale, is the same as within the zone of capture. Effective sampling area is further multiplied by 50 m, representing the area of the water column in which most krill reside and moult (see above), to derive the effective sampling volume, V (m^3) which is 0.5·50.

Through combining all parameterisations to this point, we can calculate mean krill concentration, K_{mean} (ind. m^{-3}), and mean biomass, B_{mean} (g m^{-3}), within the mean spatial scale of the sediment trap for each sample bottle period, j as follows:

$$K_{j,mean} = \overline{IMP_j} \cdot e_j \cdot V^{-1} \quad (5)$$

$$B_{j,mean} = G_j \cdot V^{-1} \quad (6)$$

For each sediment trap bottle j, an estimate of variability in mean krill concentration or biomass was obtained by deriving the sample standard deviation (SD) of krill concentration calculated using stage-specific IMP data for each krill (Section 1.2).

$K_{j,mean}$ and $B_{j,mean}$ values were converted into units of m^{-2} through multiplying by 50 m (the surface depth interval within which all krill were assumed to reside during nighttime, when moulting was assumed to take place; supplementary information table 2.1 and 2.2).

5. Deriving reasonable upper and lower bounds around the mean estimate

To derive upper and lower bounds around the mean estimate, we must revisit the assumption that the distribution of krill is even at the 1 to 2 km spatial scale of capture over a 15 or 30 day sampling interval. The alternative scenario is that the distribution is uneven to the extent that the captured number of exuviae either over-represents or under-represents krill biomass at this spatial scale. In order to calculate this, we require an estimate of unevenness at an appropriate spatial scale. We refer to acoustic transect estimates of krill distribution and biomass carried out as part of the Western Core Box time series (www.bas.ac.uk/project/poets-wcb/), which geographically encompasses the location of the present sediment trap mooring. That data series partitions transects into spatial cells of 500 m length. Fielding et al. (2014) estimated the Coefficient of variation (CV, %) between these cells for different years, spanning 1997 to 2013. It is important to note that these estimates were made in the same season each year (spring-summer) and do not have seasonal coverage. Nevertheless, for this region, they provide the best estimate available of how krill distribution varies on a scale relevant to the present spatial scale of capture.

At a cell size of 500 m, there may be between 2 and 4 cells within the present spatial scale of capture of the sediment trap, making the resolution with which to assess variability in distribution reasonable for present purposes. Fielding et al. (2014; their supplementary information table 2.2, SDWBA 3 frequency method) report CV values that range between ~10 and ~60% (95% percentile value of 58.6%). Accordingly, biomass within a cell from where the majority of exuviae enter the zone of capture may be up to ~60% higher or lower than other neighbouring cells, so resulting in an overestimate or underestimate respectively of true biomass. Such a scenario further assumes that this same uneven distribution between cells persists for the duration of the sediment trap collection interval of 15 or 30 days, which is unlikely. Nevertheless, assuming the temporal persistence of this unevenness allows us to calculate reasonable outer bounds for the mean biomass estimate calculated above.

To calculate the outer bounds, we assumed that the captured number of exuviae were either an under- or over-estimate of the abundance of krill in the spatial scale of capture. The number of captured exuviae in bottle *j* was therefore increased or decreased by the 95% percentile CV ($cv_{95,}$ 58.6%) from Fielding et al. (2014) as follows:

$$K_{j,outer\pm} = \overline{IMP_j} \cdot \left(e_j.(1 \pm cv_{95})\right) \cdot V^{-1} \qquad (7)$$

$$B_{j,outer\pm} = \frac{\sum_{i=1}^{i=m} \overline{IMP_{i,j}} \cdot w_{i,j}}{m_j} \cdot \left(e_j.(1 \pm cv_{95})\right) \cdot V^{-1} \qquad (8)$$

Estimates of SD were calculated as for Eq. 5 and 6. Values derived for $K_{j,outer\pm}$ and $B_{j,outer\pm}$ are presented in Supplementary information tables 2.2 and 2.3.

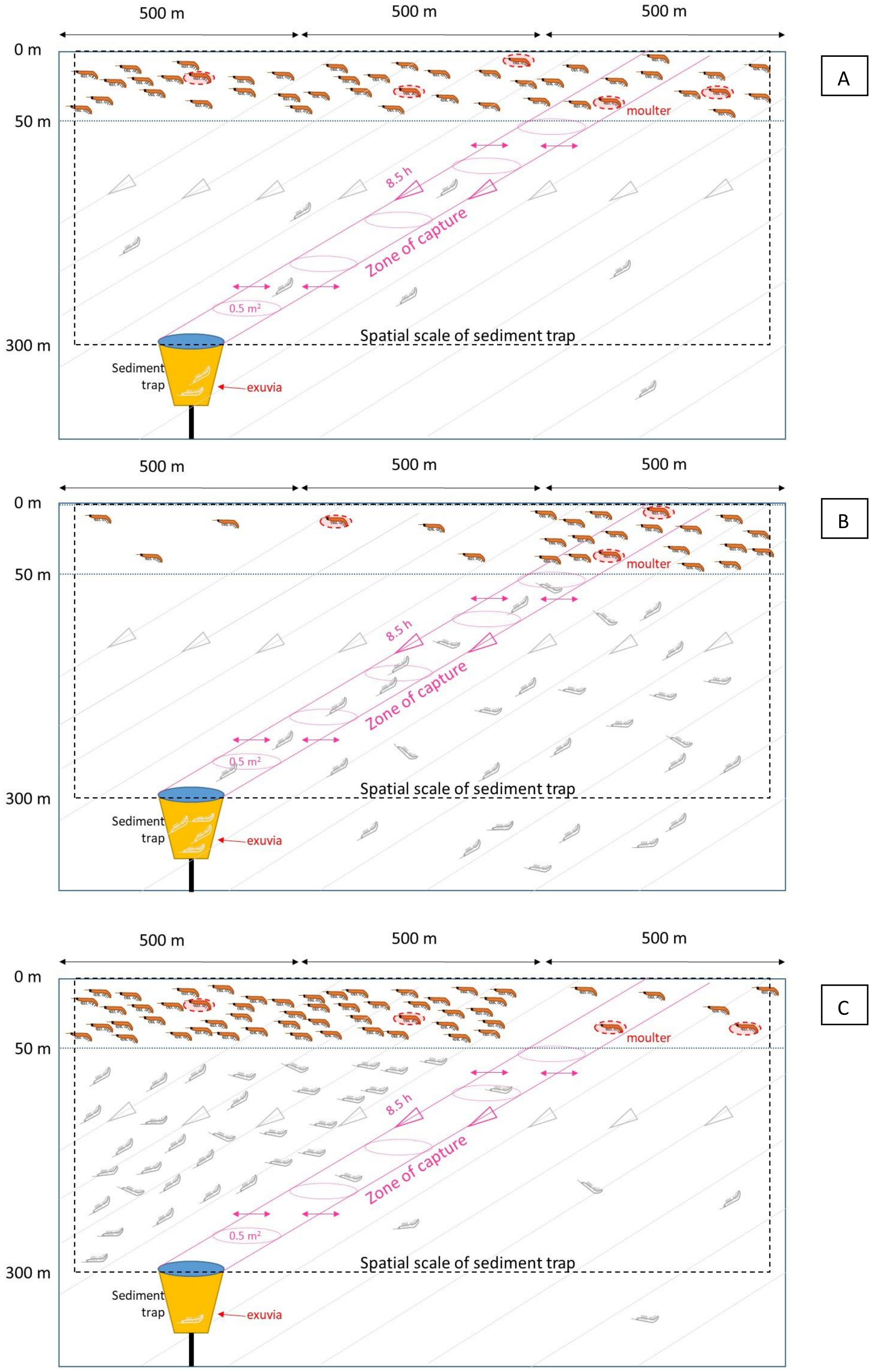
500 m
500 m
500 m
0 m
50 m
300 m
A
moulter
8.5 h
Zone of capture
0.5 m²
Spatial scale of sediment trap
Sediment trap
exuvia
500 m
500 m
500 m
0 m
50 m
300 m
B
moulter
8.5 h
Zone of capture
0.5 m²
Spatial scale of sediment trap
Sediment trap
exuvia
500 m
500 m
500 m
0 m
50 m
300 m
C
moulter
8.5 h
Zone of capture
0.5 m²
Spatial scale of sediment trap
Sediment trap
exuvia

Supplementary information Figure 1: Potential scenarios of krill distribution during a daily sampling period (starting from the assumed point of peak moulting at midnight), A: where the distribution was even over the spatial scale of collection (i.e. the distance between the sediment trap and the remote location from which a captured exuvia was released, which was between 1 and 2 km on average), B: where there was an over-representation of krill in the area around the zone of capture and C: where there was an under-representation in the area around the zone of capture. In Scenario A, the moults captured by the sediment trap can be considered to represent the mean situation within the spatial scale of sampling, while they over-represent and under-represent the true situation at this spatial scale in Scenarios B and C respectively. The 500 m zones mark the size of the cells for which biomass was assessed as part of acoustic transects carried out historically in this region (Fielding et al. 2014). That study estimated the coefficient of variance in biomass between cells to be up to 60%.

6. Calculation of krill-exuvia carbon flux at the Southern Ocean scale

In order to scale up the exuvia carbon flux calculated from the sediment trap data to the Southern Ocean krill population, we assumed a population biomass of Antarctic krill in the Southern Ocean (P) of 379 Mt (wet weight [WW] biomass). The exuvia flux per sediment trap bottle (Fj, g C m-2 d-1) and corresponding krill biomass (Bj, g WW m-2) are provided in Supplementary information Table 2.1 and Table 2.3 respectively (note we have converted Fj from mg to g for the present calculation). For each g WW of krill, the corresponding exuvia flux (g C d-1) is Fj · Bj-1. Exuvia flux for the Southern Ocean population of krill was determined as P · Fj · Bj-1 t C d-1. At the Southern Ocean scale, the estimate exuvia flux varied according to bottle j, with a mean of 0.29 Mt C d-1 (SE 0.09) and range of 0.07 Mt C d-1 to 1.63 Mt C d-1 (excluding September where no exuviae were captured; Table S2.4). The main factors causing the variation were the estimate of IMP, which varied according to standard length and temperature (sex/stage was not possible to determine), and the ratio of IMP to Bj, which varied according to the population structure.

Sediment trap bottle period (j)	15 Jan - 30 Jan 2017	30 Jan - 14 Feb 2017	14 Feb – 1 Mar 2017	1 Mar - 31 Mar 2017	31 Mar - 30 Apr 2017	30 Apr - 30 May 2017	30 May - 29 Jun 2017	29 Jun - 29 Jul 2017
Number of days (d)	15	15	15	30	30	30	30	30
Mean daily distance ($D_{j,mean}$, m)	1403	1633	1359	1713	1610	1510	1082	1698
Minimum daily distance ($D_{j,min}$, m)	596	459	532	432	79	415	283	284
Maximum daily distance ($D_{j,max}$, m)	3200	3546	2251	4060	3190	3843	3277	3322
SD (m)	601	870	503	903	803	753	589	802

Sediment trap bottle period (j)	29 Jul - 28 Aug 2017	28 Aug - 27 Sep 2017	27 Sep - 27 Oct 2017	27 Oct - 11 Nov 2017	11 Nov - 26 Nov 2017	26 Nov - 11 Dec 2017	11 Dec - 26 Dec 2017	26 Dec 2017 - 6 Jan 2018
Number of days (d)	30	30	30	15	15	15	15	12
Mean daily distance ($D_{j,mean}$, m)	1769	1456	1147	1491	1089	1346	1330	1392
Minimum daily distance ($D_{j,min}$, m)	335	213	192	677	166	435	385	207
Maximum daily distance ($D_{j,max}$, m)	4692	3517	2863	2532	2116	2253	2570	2672
SD (m)	1112	837	648	545	655	527	589	659

Supplementary information Table 2.1: Straight distance between the location of the sediment trap and the starting location of the exuviae, assuming a sinking speed of 1 cm s^{-1} from the surface. Calculations based on horizontal velocity data from a moored upward looking 300 kHz ADCP situated above the sediment trap. Note that the ADCP record ended before the final sediment trap bottle closed, hence a shorter time period was used for the ADCP data analysis for the final bottle than for the krill calculations.

Sediment trap bottle period (*j*)		15 Jan - 30 Jan 2017	30 Jan - 14 Feb 2017	14 Feb - 1 Mar 2017	1 Mar - 31 Mar 2017	31 Mar - 30 Apr 2017	30 Apr - 30 May 2017	30 May - 29 Jun 2017	29 Jun - 29 Jul 2017
Number of days (*d*)		15	15	15	30	30	30	30	30
Number of exuviae in bottle *j*		280	512	210	100	4	4	4	3
Seawater temperature (Whitehouse et al. 2008)		2.93	3.23	3.37	3.41	3.2	2.61	1.88	1.3
Krill concentration ($K_{j,mean}$, ind m^{-2})	Mean	538.049	1005.187	405.894	91.507	18.995	18.116	18.546	13.470
SD (ind m^{-2})		113.328	189.760	80.197	26.048	4.052	4.920	4.642	3.338
Krill concentration ($K_{j,outer+}$, ind m^{-2})	Upper bound	853.345	1594.226	643.748	145.131	30.126	28.731	29.415	21.364
SD (ind m^{-2})		179.739	300.960	127.192	41.312	6.427	7.802	7.361	5.294
Krill concentration ($K_{j,outer-}$, ind m^{-2})	Lower bound	222.752	416.147	168.040	37.884	7.864	7.500	7.678	5.577
SD (ind m^{-2})		46.918	78.561	33.201	10.784	1.678	2.037	1.922	1.382

Sediment trap bottle period (j)		29 Jul - 28 Aug 2017	28 Aug - 27 Sep 2017	27 Sep - 27 Oct 2017	27 Oct - 11 Nov 2017	11 Nov - 26 Nov 2017	26 Nov - 11 Dec 2017	11 Dec - 26 Dec 2017	26 Dec 2017 - 9 Jan 2018
Number of days (d)		30	30	30	15	15	15	15	14
Number of exuviae in bottle *j*		2	0	3	3	3	23	27	94
Seawater temperature (Whitehouse et al. 2008)		0.49	0.27	0.46	0.98	1.29	1.71	2	2.56
Krill concentration ($K_{j,mean}$, ind m^{-2})	Mean	9.132	0	2.741	5.983	5.482	43.145	49.996	189.798
SD (ind m^{-2})		2.739	0	0.885	1.076	1.354	9.499	11.656	43.660
Krill concentration ($K_{j,outer+}$, ind m^{-2})	Upper bound	14.483	0	4.348	9.489	8.694	68.429	79.294	301.020
SD (ind m^{-2})		4.344	0	1.403	1.707	2.147	15.066	18.487	69.246
Krill concentration ($K_{j,outer-}$, ind m^{-2})	Lower bound	3.781	0	1.135	2.477	2.269	17.862	20.698	78.576
SD (ind m^{-2})		1.134	0	0.366	0.445	0.560	3.933	4.826	18.075

Supplementary informationTable 2.2: Estimates of the concentration of krill (ind m^{-2}) during sediment trap deployment in the northern Scotia Sea during 2017-2018. Red denotes winter period where IMP was assumed to increase by a factor of 5

Sediment trap bottle period (j)		15 Jan - 29 Jan 2017	30 Jan - 13 Feb 2017	14 Feb - 28 Feb 2017	1 March - 30 March 2017	31 March -29 April 2017	30 April - 29 May 2017	30 May - 28 Jun 2017	29 Jun - 28 Jul 2017
Number of days (d)		15	15	15	30	30	30	30	30
Number of exuviae in bottle *j*		280	512	210	100	4	4	4	3
Seawater temperature (Whitehouse et al. 2008)		2.93	3.23	3.37	3.41	3.2	2.61	1.88	1.3
Krill biomass ($W_{j,mean}$, g m^{-2})	Mean	130.095	260.936	85.794	9.693	3.572	2.479	4.736	2.766
SD (g m^{-2})		78.868	147.790	45.639	7.412	1.385	1.103	3.880	0.721
Krill biomass ($W_{j,outer+}$, g m^{-2})	Upper bound	206.331	413.844	136.070	15.373	5.665	3.932	7.512	4.387
SD (g m^{-2})		125.084	234.395	72.384	11.755	2.197	1.749	6.154	1.144
Krill biomass ($W_{j,outer-}$, g m^{-2})	Lower bound	53.859	108.027	35.519	4.013	1.479	1.026	1.961	1.145
SD (g m^{-2})		32.651	61.185	18.895	3.068	0.574	0.457	1.606	0.299

Sediment trap bottle period (j)		29 Jul - 27 Aug 2017	28 Aug - 26 Sep 2017	27 Sep - 26 Oct 2017	27 Oct - 10 Nov 2017	11 Nov - 25 Nov 2017	26 Nov - 10 Dec 2017	11 Dec - 25 Dec 2017	26 Dec 2017 - 9 Jan 2018
Number of days (d)		30	30	30	15	15	15	15	14
Number of exuviae in bottle *j*		2	0	3	3	3	23	27	94
Seawater temperature (Whitehouse et al. 2008)		0.49	0.27	0.46	0.98	1.29	1.71	2	2.56
Krill biomass ($W_{j,mean}$, g m^{-2})	Mean	2.459	0	0.756	3.077	1.404	12.684	11.826	44.018
SD (g m^{-2})		1.262	0	0.620	1.216	0.862	7.615	7.238	29.628
Krill biomass ($W_{j,outer+}$, g m^{-2})	Upper bound	3.900	0	1.198	4.880	2.227	20.117	18.756	69.813
SD (g m^{-2})		2.001	0	0.984	1.929	1.367	12.078	11.479	46.990
Krill biomass ($W_{j,outer-}$, g m^{-2})	Lower bound	1.018	0	0.313	1.274	0.581	5.251	4.896	18.224
SD (g m^{-2})		0.522	0	0.257	0.503	0.357	3.153	2.996	12.266

Supplementary information Table 2.3: Estimates of the biomass of krill (g m^{-2}) during sediment trap deployment in the northern Scotia Sea during 2017-2018. Red denotes winter period where IMP was assumed to increase by a factor of 5

Sediment trap bottle period (*j*)		15 Jan - 30 Jan 2017	30 Jan - 14 Feb 2017	14 Feb - 1 Mar 2017	1 Mar - 31 Mar 2017	31 Mar - 30 Apr 2017	30 Apr - 30 May 2017	30 May - 29 Jun 2017	29 Jun - 29 Jul 2017
Exuvia flux (*F*)	mg C m^{-2} d^{-1}	59.73	210.70	63.80	38.67	0.61	0.61	1.12	0.88
WW biomass (*B*)	g WW m^{-2}	130.10	260.94	85.79	9.69	3.57	2.48	4.74	2.77
Weight specific (g WW^{-1}) exuvia flux	g C d^{-1}	0.49	0.86	0.80	4.29	0.17	0.25	0.24	0.32
Exuvia flux for 379 Mt of krill	Mt C d^{-1}	0.19	0.33	0.30	1.63	0.07	0.09	0.09	0.12

Sediment trap bottle period (j)		29 Jul - 28 Aug 2017	28 Aug - 27 Sep 2017	27 Sep - 27 Oct 2017	27 Oct - 11 Nov 2017	11 Nov - 26 Nov 2017	26 Nov - 11 Dec 2017	11 Dec - 26 Dec 2017	26 Dec 2017 - 9 Jan 2018	**Seasonal Mean (SE)**
Exuvia flux (*F*)	mg C m^{-2} d^{-1}	0.53	0.00	0.84	1.84	0.84	6.44	7.92	41.08	**27.23 (13.46)**
WW biomass (*B*)	g WW m^{-2}	2.46	0.00	0.76	3.08	1.40	12.68	11.83	44.02	**36.02 (17.56)**
Weight specific exuvia flux	g C d^{-1}	0.22	0.00	1.20	0.64	0.65	0.55	0.72	1.00	**0.78 (0.25)**
Exuvia flux for 379 Mt of krill	Mt C d^{-1}	0.08	0.00	0.45	0.24	0.24	0.21	0.27	0.38	**0.29 (0.09)**

Supplementary information Table 2.4: Estimates of the total daily exuvia flux for Southern Ocean biomass of Antarctic krill using parameterisations from a sediment trap deployment in the northern Scotia Sea during 2017-2018 and assuming a Southern Ocean biomass (*P*) of 379 Mt (Atkinson et al. 2009).

Supplementary References:

Atkinson A, Siegel V, Pakhomov EA, Jessopp MJ, Loeb V (2009) A re-appraisal of the total biomass and annual production of Antarctic krill. Deep-Sea Res Part I-Oceanogr Res Pap 56: 727-740

Brierley AS, Saunders RA, Bone DG, Murphy EJ, Enderlein P, Conti SG, Demer DA (2006) Use of moored acoustic instruments to measure short-term variability in abundance of Antarctic krill. Limnology and Oceanography Methods 4: 18-29

Buchholz F, Morris DJ, Watkins JL (1989) Analyses of field moult data: prediction of intermoult period and assessment of seasonal growth in Antarctic krill, *Euphausia superba* Dana. Antarct Sci 1: 301-306

Fielding, S. et al. Interannual variability in Antarctic krill (Euphausia superba) density at South Georgia, Southern Ocean: 1997–2013, ICES J. Mar. Sci. 71 (9), 2578–2588 (2014). doi:10.1093/icesjms/fsu104

Kils U (1981) Swimming behaviour, swimming performance and energy balance of Antarctic krill *Euphausia superba*. BIOMASS Scientific Series 3: pp. 1-121

Miller D (1983) Variation in body length measurement of Euphausia superba Dana. Polar Biol 2: 17-20

Nicol S, Stolp M (1989) Sinking rates of cast exoskeletons of Antarctic krill (Euphausia superba Dana) and their role in the vertical flux of particulate matter and fluoride in the Southern Ocean. Deep Sea Research Part A Oceanographic Research Papers 36: 1753-1762

Tarling GA, Cuzin-Roudy J, Buchholz F (1999) Vertical migration behaviour in the northern krill *Meganyctiphanes norvegica* is influenced by moult and reproductive processes. Mar Ecol Prog Ser 190: 253-262 doi 10.3354/meps190253

Tarling GA, Klevjer T, Fielding S, Watkins J, Atkinson A, Murphy E, Korb R, Whitehouse M, Leaper R (2009) Variability and predictability of Antarctic krill swarm structure. Deep-Sea Res Part I-Oceanogr Res Pap 56: 1994-2012 doi 10.1016/j.dsr.2009.07.004

Tarling GA, Shreeve RS, Hirst AG, Atkinson A, Pond DW, Murphy EJ, Watkins JL (2006) Natural growth rates in Antarctic krill (*Euphausia superba*): I. Improving methodology and predicting intermolt period. Limnol Oceanogr 51: 959-972

Whitehouse MJ, Meredith MP, Rothery P, Atkinson A, Ward P, Korb RE (2008) Rapid warming of the ocean around South Georgia, Southern Ocean, during the 20th century: Forcings, characteristics and implications for lower trophic levels. Deep - Sea Research Part I - Oceanographic Research Papers 55: 1218-1228
