# Supplementary material for: Continuous moulting by Antarctic krill drives major pulses of carbon export in the north Scotia Sea, Southern Ocean
Source: Nat Commun. 2020 Nov 27;11:6051. doi: 10.1038/s41467-020-19956-7 (PMC7699634; doi:10.1038/s41467-020-19956-7)
Supplement: Supplementary file 3 — Supplementary Data 1 [file 41467_2020_19956_MOESM3_ESM.docx]

Supplementary material for **Continuous moulting by Antarctic krill drives major pulses of carbon export in the north Scotia Sea, Southern Ocean** *by Manno et al.*

1. Data set including POC, krill C contribution, uropod length and krill stock estimation

|  | **POC** | **FP** | **EXU** | **CARC** | **SD POC** | **SD**  **FP** | **SD EXU** | **SD CARC** |
| --- | --- | --- | --- | --- | --- | --- | --- | --- |
| **JAN** | 106.11 | 46.39 | 59.73 | 0 | 21.22 | 6.78 | 5.67 | 0 |
| **FEB** | 463.86 | 252.40 | 210.70 | 0 | 54.54 | 24.56 | 22.32 | 0 |
| **FEB** | 116.76 | 49.11 | 63.80 | 3.21 | 18.76 | 8.90 | 8.90 | 3.34 |
| **MAR** | 138.38 | 97.55 | 38.67 | 2.49 | 20.65 | 8.96 | 10.23 | 4.11 |
| **APR** | 5.56 | 1.11 | 0.61 | 3.98 | 1.23 | 0.34 | 0.56 | 3.67 |
| **MAY** | 6.85 | 1.40 | 0.61 | 4.98 | 1.34 | 1.23 | 0.78 | 4.03 |
| **JUN** | 4.29 | 0.76 | 1.12 | 2.30 | 2.21 | 0.34 | 1.01 | 4.34 |
| **JUL** | 3.81 | 0.47 | 0.88 | 2.45 | 1.34 | 0.25 | 0.99 | 3.99 |
| **AUG** | 3.62 | 0.07 | 0.53 | 3.00 | 1.23 | 0.07 | 0.45 | 3.45 |
| **SEP** | 3.94 | 0.10 | 0 | 3.90 | 0.65 | 0.10 | 0 | 2.67 |
| **OCT** | 2.87 | 0.10 | 0.84 | 1.93 | 1.65 | 0.14 | 1.03 | 3.45 |
| **OCT** | 4.48 | 0.50 | 1.84 | 2.13 | 2.65 | 0.14 | 1.23 | 4.01 |
| **NOV** | 3.08 | 0.08 | 0.84 | 2.16 | 2.45 | 0.12 | 0.34 | 4.11 |
| **NOV** | 12.40 | 1.16 | 6.44 | 0 | 3.45 | 1.23 | 2.34 | 0 |
| **DEC** | 15.42 | 1.51 | 7.92 | 0 | 4.56 | 1.67 | 2.45 | 0 |
| **DEC** | 109.45 | 68.50 | 41.08 | 0 | 25.67 | 10.11 | 6.78 | 0 |

**Supplementary table 1.1** Data set of Particulate Organic carbon (POC) flux and the contribution to the POC flux by krill Faecal Pellets (FPs), Exuviae (EXU) and Carcasses (CARC). Data are expressed as mg C m^-2^ d^-1^, at WCB mooring station, South Georgia Island, Scotia Sea, during January 2017-January 2018.

| **Length (mm)** | **JAN** | **FEB** | **FEB** | **MAR** | **APR** | **MAY** | **JUN** | **JUL** | **AUG** |
| --- | --- | --- | --- | --- | --- | --- | --- | --- | --- |
| **1** | 4.22 | 3.23 | 3.87 | 5.54 | 5.02 | 3.55 | 3.82 | 4.70 | 5.66 |
| **2** | 4.09 | 5.76 | 3.01 | 3.94 | 4.53 | 3.88 | 4.51 | 4.38 | 4.17 |
| **3** | 3.89 | 5.40 | 3.12 | 5.00 | 4.05 | 4.53 | 6.48 | 4.51 |  |
| **4** | 3.40 | 6.18 | 5.23 | 4.95 | 3.64 | 3.16 | 3.53 |  |  |
| **5** | 5.68 | 5.80 | 3.85 | 6.06 |  |  |  |  |  |
| **6** | 5.18 | 4.67 | 4.18 | 5.11 |  |  |  |  |  |
| **7** | 3.54 | 5.55 | 3.46 | 3.48 |  |  |  |  |  |
| **8** | 4.13 | 5.80 | 3.12 | 3.36 |  |  |  |  |  |
| **9** | 4.40 | 5.85 | 5.00 | 4.32 |  |  |  |  |  |
| **10** | 3.79 | 4.31 | 4.50 | 3.14 |  |  |  |  |  |
| **11** | 5.42 | 3.12 | 4.18 | 5.11 |  |  |  |  |  |
| **12** | 4.61 | 5.59 | 4.94 | 4.14 |  |  |  |  |  |
| **13** | 5.90 | 3.98 | 5.41 | 6.33 |  |  |  |  |  |
| **14** | 5.06 | 5.87 | 5.58 | 3.49 |  |  |  |  |  |
| **15** | 3.98 | 3.60 | 3.20 | 5.31 |  |  |  |  |  |
| **16** | 4.43 | 3.72 | 4.06 | 5.25 |  |  |  |  |  |
| **17** | 5.01 | 5.81 | 3.96 | 5.72 |  |  |  |  |  |
| **18** | 4.03 | 3.40 | 4.14 | 5.99 |  |  |  |  |  |
| **19** | 3.46 | 5.31 | 3.91 | 3.17 |  |  |  |  |  |
| **20** | 3.16 | 4.68 | 3.75 | 5.15 |  |  |  |  |  |
| **21** | 4.18 | 5.35 | 4.25 | 4.20 |  |  |  |  |  |
| **22** | 3.10 | 4.05 | 3.45 | 5.16 |  |  |  |  |  |
| **23** | 5.70 | 5.69 | 3.88 | 4.04 |  |  |  |  |  |
| **24** | 6.14 | 5.24 | 3.36 | 3.46 |  |  |  |  |  |
| **25** | 5.91 | 4.04 | 3.14 | 6.30 |  |  |  |  |  |
| **26** | 6.36 | 3.26 | 5.15 | 3.50 |  |  |  |  |  |
| **27** | 6.41 | 5.00 | 4.90 | 3.40 |  |  |  |  |  |
| **28** | 4.76 | 3.69 | 4.74 | 4.44 |  |  |  |  |  |
| **29** | 5.55 | 4.18 | 4.39 | 5.39 |  |  |  |  |  |
| **30** | 3.65 | 6.39 | 5.01 | 5.56 |  |  |  |  |  |
| **31** | 5.89 | 4.84 | 5.51 | 5.80 |  |  |  |  |  |
| **32** | 4.27 | 4.93 | 5.99 | 4.98 |  |  |  |  |  |
| **33** | 3.46 | 6.25 | 5.34 | 6.38 |  |  |  |  |  |
| **34** | 3.61 | 4.68 | 4.37 | 6.39 |  |  |  |  |  |
| **35** | 4.08 | 5.03 | 5.41 | 3.10 |  |  |  |  |  |
| **36** | 5.08 | 3.55 | 3.05 | 5.79 |  |  |  |  |  |
| **37** | 4.05 | 4.90 | 5.06 | 4.43 |  |  |  |  |  |
| **38** | 4.80 | 3.60 | 4.73 | 4.30 |  |  |  |  |  |
| **39** | 3.60 | 5.26 | 3.95 | 5.56 |  |  |  |  |  |
| **40** | 6.29 | 3.48 | 3.38 | 3.00 |  |  |  |  |  |
| **41** | 5.63 | 3.88 | 5.69 | 4.95 |  |  |  |  |  |
| **42** | 5.62 | 4.10 | 5.91 | 3.42 |  |  |  |  |  |
| **43** | 5.00 | 3.83 | 4.83 | 4.18 |  |  |  |  |  |
| **44** | 4.42 | 6.31 | 3.90 | 4.64 |  |  |  |  |  |
| **45** | 3.73 | 4.71 | 4.19 | 5.84 |  |  |  |  |  |
| **46** | 6.27 | 3.60 | 5.67 | 4.77 |  |  |  |  |  |
| **47** | 4.80 | 5.99 | 4.10 | 5.01 |  |  |  |  |  |
| **48** | 3.99 | 5.59 | 4.42 | 6.04 |  |  |  |  |  |
| **49** | 3.24 | 3.61 | 4.48 | 3.12 |  |  |  |  |  |
| **50** | 3.60 | 3.28 | 4.78 | 6.30 |  |  |  |  |  |
| **51** | 5.91 | 4.82 | 5.13 | 4.01 |  |  |  |  |  |
| **52** | 5.54 | 3.31 | 4.03 | 5.78 |  |  |  |  |  |
| **53** | 4.26 | 5.49 | 4.86 | 4.85 |  |  |  |  |  |
| **54** | 3.05 | 4.12 | 4.66 | 4.80 |  |  |  |  |  |
| **55** | 3.24 | 3.71 | 4.03 | 5.74 |  |  |  |  |  |
| **56** | 3.24 | 4.04 | 5.92 | 3.96 |  |  |  |  |  |
| **57** | 4.84 | 4.82 | 4.73 | 4.26 |  |  |  |  |  |
| **58** | 5.11 | 5.80 | 5.42 | 5.57 |  |  |  |  |  |
| **59** | 4.78 | 3.66 | 4.03 | 4.10 |  |  |  |  |  |
| **60** | 3.62 | 4.88 | 4.61 | 3.70 |  |  |  |  |  |
| **61** | 5.36 | 6.20 | 4.07 | 5.17 |  |  |  |  |  |
| **62** | 6.41 | 5.04 | 4.97 | 4.87 |  |  |  |  |  |
| **63** | 5.94 | 6.22 | 3.44 | 4.48 |  |  |  |  |  |
| **64** | 5.55 | 4.50 | 5.11 | 6.36 |  |  |  |  |  |
| **65** | 3.43 | 3.68 | 4.63 | 4.47 |  |  |  |  |  |
| **66** | 4.33 | 6.02 | 5.23 | 3.51 |  |  |  |  |  |
| **67** | 3.66 | 5.59 | 3.81 | 5.59 |  |  |  |  |  |
| **68** | 5.10 | 3.33 | 3.10 | 4.70 |  |  |  |  |  |
| **69** | 5.55 | 3.89 | 5.80 | 4.90 |  |  |  |  |  |
| **70** | 5.76 | 6.01 | 5.42 | 3.00 |  |  |  |  |  |
| **71** | 4.78 | 4.88 | 3.83 | 3.29 |  |  |  |  |  |
| **72** | 4.90 | 5.65 | 3.98 | 6.24 |  |  |  |  |  |
| **73** | 4.71 | 5.59 | 5.77 | 5.80 |  |  |  |  |  |
| **74** | 3.34 | 5.41 | 3.33 | 5.11 |  |  |  |  |  |
| **75** | 5.83 | 6.12 | 5.90 | 4.29 |  |  |  |  |  |
| **76** | 3.14 | 4.30 | 5.83 | 3.53 |  |  |  |  |  |
| **77** | 3.65 | 3.15 | 3.36 | 4.02 |  |  |  |  |  |
| **78** | 4.03 | 5.28 | 4.25 | 5.18 |  |  |  |  |  |
| **79** | 6.20 | 5.56 | 4.98 | 3.03 |  |  |  |  |  |
| **80** | 5.89 | 5.28 | 4.70 | 4.52 |  |  |  |  |  |
| **81** | 3.42 | 4.81 | 4.05 |  |  |  |  |  |  |
| **82** | 3.16 | 4.40 | 3.99 |  |  |  |  |  |  |
| **83** | 3.75 | 4.13 | 3.24 |  |  |  |  |  |  |
| **84** | 5.76 | 3.08 | 4.57 |  |  |  |  |  |  |
| **85** | 4.83 | 6.26 | 5.84 |  |  |  |  |  |  |
| **86** | 4.51 | 6.33 | 4.71 |  |  |  |  |  |  |
| **87** | 5.37 | 4.90 | 3.75 |  |  |  |  |  |  |
| **88** | 3.44 | 6.17 | 4.50 |  |  |  |  |  |  |
| **89** | 5.43 | 5.02 | 3.98 |  |  |  |  |  |  |
| **90** | 3.91 | 3.64 | 3.73 |  |  |  |  |  |  |
| **91** | 3.67 | 5.64 | 3.98 |  |  |  |  |  |  |
| **92** | 4.11 | 3.56 | 5.48 |  |  |  |  |  |  |
| **93** | 5.29 | 4.28 | 3.39 |  |  |  |  |  |  |
| **94** | 3.38 | 3.62 | 5.11 |  |  |  |  |  |  |
| **95** | 5.80 | 3.85 | 3.42 |  |  |  |  |  |  |
| **96** | 4.82 | 4.45 | 4.10 |  |  |  |  |  |  |
| **97** | 3.12 | 4.27 | 3.41 |  |  |  |  |  |  |
| **98** | 4.64 | 4.16 | 4.00 |  |  |  |  |  |  |
| **99** | 5.73 | 5.61 | 4.37 |  |  |  |  |  |  |
| **100** | 3.90 | 3.61 | 3.65 |  |  |  |  |  |  |

| **Length (mm)** | **OCT** | **OCT** | **NOV** | **NOV** | **DEC** | **DEC** |
| --- | --- | --- | --- | --- | --- | --- |
| **1** | 3.41 | 5.20 | 4.67 | 5.50 | 4.09 | 6.24 |
| **2** | 6.44 | 7.00 | 5.95 | 4.69 | 3.02 | 6.31 |
| **3** | 4.23 | 6.69 | 3.52 | 3.33 | 5.40 | 6.38 |
| **4** |  |  |  | 6.39 | 4.19 | 3.80 |
| **5** |  |  |  | 4.17 | 3.55 | 4.52 |
| **6** |  |  |  | 6.31 | 6.17 | 4.18 |
| **7** |  |  |  | 5.02 | 3.70 | 4.90 |
| **8** |  |  |  | 6.17 | 5.40 | 4.43 |
| **9** |  |  |  | 4.52 | 4.84 | 4.16 |
| **10** |  |  |  | 3.39 | 4.16 | 3.58 |
| **11** |  |  |  | 5.97 | 4.31 | 4.10 |
| **12** |  |  |  | 4.91 | 5.44 | 4.93 |
| **13** |  |  |  | 3.52 | 4.09 | 3.00 |
| **14** |  |  |  | 5.23 | 6.19 | 6.27 |
| **15** |  |  |  | 5.74 | 3.45 | 5.42 |
| **16** |  |  |  | 6.34 | 5.54 | 3.55 |
| **17** |  |  |  | 3.17 | 3.15 | 6.10 |
| **18** |  |  |  | 4.05 | 3.85 | 3.18 |
| **19** |  |  |  | 6.32 | 4.90 | 4.23 |
| **20** |  |  |  | 4.10 | 5.82 | 3.42 |
| **21** |  |  |  |  |  | 4.97 |
| **22** |  |  |  |  |  | 3.03 |
| **23** |  |  |  |  |  | 3.97 |
| **24** |  |  |  |  |  | 6.42 |
| **25** |  |  |  |  |  | 3.80 |
| **26** |  |  |  |  |  | 3.57 |
| **27** |  |  |  |  |  | 4.56 |
| **28** |  |  |  |  |  | 4.52 |
| **29** |  |  |  |  |  | 3.56 |
| **30** |  |  |  |  |  | 5.50 |
| **31** |  |  |  |  |  | 6.26 |
| **32** |  |  |  |  |  | 5.67 |
| **33** |  |  |  |  |  | 4.90 |
| **34** |  |  |  |  |  | 5.23 |
| **35** |  |  |  |  |  | 3.58 |
| **36** |  |  |  |  |  | 3.57 |
| **37** |  |  |  |  |  | 3.04 |
| **38** |  |  |  |  |  | 5.72 |
| **39** |  |  |  |  |  | 3.43 |
| **40** |  |  |  |  |  | 5.59 |
| **41** |  |  |  |  |  | 5.67 |
| **42** |  |  |  |  |  | 3.34 |
| **43** |  |  |  |  |  | 6.31 |
| **44** |  |  |  |  |  | 3.65 |
| **45** |  |  |  |  |  | 6.37 |
| **46** |  |  |  |  |  | 4.25 |
| **47** |  |  |  |  |  | 3.61 |
| **48** |  |  |  |  |  | 5.05 |
| **49** |  |  |  |  |  | 5.34 |
| **50** |  |  |  |  |  | 4.93 |
| **51** |  |  |  |  |  | 3.36 |
| **52** |  |  |  |  |  | 3.63 |
| **53** |  |  |  |  |  | 5.42 |
| **54** |  |  |  |  |  | 3.00 |
| **55** |  |  |  |  |  | 3.58 |
| **56** |  |  |  |  |  | 3.36 |
| **57** |  |  |  |  |  | 3.12 |
| **58** |  |  |  |  |  | 5.24 |
| **59** |  |  |  |  |  | 3.48 |
| **60** |  |  |  |  |  | 5.26 |
| **61** |  |  |  |  |  | 3.42 |
| **62** |  |  |  |  |  | 4.01 |
| **63** |  |  |  |  |  | 5.87 |
| **64** |  |  |  |  |  | 4.45 |
| **65** |  |  |  |  |  | 3.78 |
| **66** |  |  |  |  |  | 4.65 |
| **67** |  |  |  |  |  | 3.13 |
| **68** |  |  |  |  |  | 4.30 |
| **69** |  |  |  |  |  | 5.21 |
| **70** |  |  |  |  |  | 4.44 |
| **71** |  |  |  |  |  | 3.33 |
| **72** |  |  |  |  |  | 4.96 |
| **73** |  |  |  |  |  | 4.23 |
| **74** |  |  |  |  |  | 5.56 |
| **75** |  |  |  |  |  | 6.07 |
| **76** |  |  |  |  |  | 3.87 |
| **77** |  |  |  |  |  | 3.25 |
| **78** |  |  |  |  |  | 4.67 |
| **79** |  |  |  |  |  | 4.24 |
| **80** |  |  |  |  |  | 3.98 |

**Supplementary table 1.2** Data set of krill uropod length measured from exuviae collected in the sediment trap samples deployed at WCB station, South Georgia Island, Scotia Sea, during January 2017-January 2018. Length of uropod is expressed in mm.

|  | **Krill biomass** | **SD** |
| --- | --- | --- |
| **JAN** | 130.095 | 78.868 |
| **FEB** | 260.936 | 147.79 |
| **FEB** | 85.794 | 45.639 |
| **MAR** | 9.693 | 7.412 |
| **APR** | 3.572 | 1.385 |
| **MAY** | 2.479 | 1.103 |
| **JUN** | 4.736 | 3.88 |
| **JUL** | 2.766 | 0.721 |
| **AUG** | 2.459 | 1.262 |
| **SEP** | 0 | 0 |
| **OCT** | 0.756 | 0.62 |
| **OCT** | 3.077 | 1.216 |
| **NOV** | 1.404 | 0.862 |
| **NOV** | 12.684 | 7.615 |
| **DEC** | 11.826 | 7.238 |
| **DEC** | 44.018 | 29.628 |

**Supplementary table 1.3** Data set of mean krill biomass expressed as g WW m^-2^
